# Barriers and facilitators of translating research evidence into public health policy and practice in Zambia: A scoping review

**DOI:** 10.4102/jphia.v17i1.1837

**Published:** 2026-10-21

**Authors:** Kwame Shanaube, Sandra Chilengi-Sakala, Kutha Banda, Princess Chiluba, Emmanuel Mukambo, Chama M. Kapatamoyo, Emmanuel Lesa, Mubanga Mulenga, Victor Chalwe, Godfrey Biemba, Mohammed Limbada

**Affiliations:** 1 Research Directorate, Zambart, Lusaka, Zambia; 2 School of Public Health, University of Zambia, Lusaka, Zambia; 3 National Health Research Authority, Lusaka, Zambia; 4 Seed Global Health, Lusaka, Zambia; 5 School of Medicine, Lusaka Apex Medical University, Lusaka, Zambia

**Keywords:** knowledge translation, policy implementation, facilitators, gaps, challenges, Zambia

## Abstract

**Background:**

Knowledge translation (KT) is increasingly recognised as a critical process for bridging the persistent ‘evidence–action’ gap in health systems. Despite global efforts to promote evidence-informed decision-making, the uptake and translation of public health research remain limited.

**Aim:**

To identify barriers and facilitators of translating research evidence into public health policy and practice in Zambia.

**Setting:**

The review focused on studies that explored barriers and facilitators of evidence-informed decision-making conducted in Zambia between 2000 and 2025.

**Method:**

The scoping review followed the Preferred Reporting Items for Systematic Reviews and Meta-Analyses for Scoping Reviews and the Arksey and O’Malley methodological framework for scoping reviews. A systematic search of both peer-reviewed literature and grey sources was conducted across PubMed Central, BioMed Central and Google Scholar, supplemented by a direct Google search.

**Results:**

Out of 6116 records identified, 24 studies met the inclusion criteria. Key barriers to KT in Zambia included inadequate relevant information for decision-making, poor access to information by policymakers, limited KT capacity, insufficient funding, weak relationships between researchers and policymakers, the absence of national KT guidelines and the lack of centralised knowledge hubs. Despite these challenges, facilitators were identified as follows: the enactment of the *National Health Research Act (2013)*, appointment of provincial KT focal persons, capacity-building initiatives and availability of engagement platforms.

**Conclusion:**

Zambia has made measurable strides towards strengthening KT by ensuring research findings are effectively translated into public health policy and practice. Yet significant key barriers such as the absence of national KT guidelines and central knowledge hubs, as well as sustainable financing, continue to limit the effectiveness of these efforts.

**Contribution:**

The gaps bridged by this review underscore the significance of developing national KT guidelines and a KT framework, including their evaluation.

## Introduction

Knowledge translation (KT) is defined as a dynamic, iterative process encompassing the synthesis, exchange, monitoring and application of research evidence to inform policies, practices and programmes.^1^ Since its formal recognition during the 2004 Ministerial Summit in Mexico, KT has been widely adopted to bridge the persistent ‘evidence–action’ gap; the disconnect between what research shows and what is actually implemented or practised.^2^ The core aim of KT is to improve population health by ensuring research findings are effectively integrated into decision-making.

Globally, the translation of research evidence into public health policy and practice remains limited, especially in Africa. Research shows that it can take up to 17 years for evidence-based findings to be adopted into routine practice,^3^ a lag that undermines health outcomes and erodes returns on scientific investment.^4^ Factors contributing to the ‘evidence-action’ gap include limited institutional capacity for evidence use, poor communication between researchers and policymakers, fragmented governance structures, and insufficient funding for KT activities.^5,6^ In Africa, these challenges are exacerbated by inadequate skills for evidence synthesis, weak inter-stakeholder collaboration and competing political priorities.^7^ In low- and middle-income countries (LMICs) such as Zambia, the limited availability of KT institutional frameworks and inconsistent stakeholder engagement have further constrained the systematic use of research in policy and practice.^3^

Weak KT infrastructure results in fragmented health policies, duplication of programmatic efforts and suboptimal health interventions.^8,9^ On the other hand, evidence-informed policymaking is associated with improved health outcomes, greater efficiency in resource allocation and stronger accountability in health systems.^10^ These realities have prompted the growing global attention to KT. The World Health Organization (WHO) Strategy on Research for Health and the 2008 Bamako Call for Action both emphasised the need for evidence-informed policies and policy-informed research.^11^ More recently, in May 2025, WHO launched a Global Research Agenda on KT and Evidence-informed Policymaking, calling for stronger systems to embed evidence into health decision-making as well as investment in KT capacity at regional and national levels.^12,13^ Other regional initiatives such as the Evidence-Informed Policy Network (EVIPNet) have similarly connected researchers, policymakers and civil society in sub-Saharan Africa (SSA) around evidence use, demonstrating that structural facilitators can meaningfully accelerate progress towards better KT outcomes.^14,15^

While programmatic reports and individual studies in Zambia have documented some barriers and facilitators to KT, no scoping review has been conducted to systematically map the range, nature and consistency of these factors across different actors and contexts. Therefore, there is no consolidated evidence base to inform the development or evaluation of a national KT framework, a critical need given Zambia’s current trajectory towards institutionalising evidence-informed decision-making.

To address this gap, we conducted a scoping review aimed at identifying barriers and facilitators of translating research evidence into public health policy and practice in Zambia. Specifically, this review sought to determine the following objectives: (1) document existing evidence on KT barriers and facilitators in Zambia; (2) clarify the scope and consistency of KT challenges across studies, actors, and health domains; (3) provide an evidence-informed foundation for developing a national KT framework and its associated evaluation framework.

## Methods

The scoping review followed the Preferred Reporting Items for Systematic Reviews and Meta-Analyses extension for Scoping Reviews (PRISMA-ScR) and Arksey and O’Malley’s^14^ steps for conducting a scoping review. This combined approach was taken because it provides the essential procedural framework for conducting the review, and the PRISMA-ScR ensures the completed review is reported with transparency, completeness and reproducibility.^14,16^

### Search strategy for identifying relevant studies

We focused on studies examining barriers and facilitators of evidence-informed decision-making for public health policy and practice in Zambia. This included studies that documented challenges, successes and the translation of research evidence into policy and practice.

We conducted searches in BioMed Central, PubMed Central, Google Scholar and grey literature sources. The search period was January 2000 to October 2025, chosen to account for the scarcity of literature on KT in Zambia. Both published evidence and grey literature on research uptake were included.

The search combined Medical Subject Headings (MeSH) terms and text words using ‘AND’ and ‘OR’ Boolean operators. MeSH terms included ‘Evidence-Based Practice’[MeSH] and ‘Translational Science’[MeSH]. These were combined with text word alternatives covering key KT concepts: ‘knowledge translation,’ ‘evidence to action,’ ‘policy implementation,’ ‘research utilisation,’ ‘knowledge mobilisation,’ ‘evidence uptake’ and ‘implementation science.’ Following reviewer feedback, additional text word alternatives were incorporated, including ‘knowledge transfer,’ ‘evidence-based practice,’ ‘evidence-informed practice,’ ‘evidence dissemination’ and ‘research-to-practice.’ Terms for barriers were expanded to include ‘barriers,’ ‘obstacles’ and ‘challenges,’ and facilitator terms were expanded to include ‘facilitators,’ ‘enablers’ and ‘drivers.’ The full search string applied was:

(“knowledge translation” OR “evidence to action” OR “policy implementation” OR “research utilization” OR “knowledge mobilization” OR “evidence uptake” OR “implementation science” OR “knowledge transfer” OR “evidence-based practice” OR “evidence-informed practice” OR “evidence dissemination” OR “research-to-practice” OR “Evidence-Based Practice”[MeSH] OR “Translational Science”[MeSH]) AND (“barriers” OR “obstacles” OR “challenges” OR “facilitators” OR “enablers” OR “drivers”) AND “Zambia”.

Search results were imported into Zotero reference manager, and duplicates were removed.

### Title and abstract relevance

For the first level of screening, only the title and abstract of each citation were screened. Titles for which an abstract was not available were included for subsequent review of the full article in the data characterisation phase.

### Data characterisation

All citations deemed relevant after title and abstract screening were considered for full-text review. Two authors (P.C. and E.M.) independently screened the titles and abstracts against the eligibility criteria and objectives of this scoping review. Further appraisal assessed the methodological quality and findings before any article was included. Disagreements between the two reviewers were resolved through consensus.

A form was developed to confirm relevance and extract key characteristics, including publication details, country of study, barriers and facilitators identified, and recommendations. The 24 included studies represented a heterogeneous mix of designs. Qualitative studies formed the largest group (*n* = 9), comprising exploratory qualitative studies, interpretive and descriptive case studies, and qualitative cross-sectional designs, including one focused specifically on policy implementation. These studies offered rich contextual insight into stakeholder perceptions and experiences of KT but were subject to researcher subjectivity, and their findings may have had limited transferability beyond the specific settings and participants studied. Quantitative studies (*n* = 5) included descriptive cross-sectional surveys, a randomised field study, and cross-sectional designs, which allowed for broader generalisation within their sampled populations but were constrained by reliance on self-reported data and potential for selection and response bias. Mixed-methods studies (*n* = 2), using sequential explanatory and sequential mixed-methods designs, provided complementary depth and breadth but varied in how rigorously the qualitative and quantitative strands were integrated. Review and secondary evidence studies (*n* = 5), including a narrative review, a literature review combined with systematic facilitated workshop discussions, a document review, a reflective commentary and a descriptive review of health research priority setting processes, which contributed synthesised and programmatic perspectives, though they carried the inherent limitations of their source materials. Implementation and programmatic studies (*n* = 3), comprising two descriptive implementation studies and one quasi-experimental pre-post intervention study, offered evidence on real-world KT efforts.

### Study selection

Study selection and management were conducted using Zotero. The original search yielded 6116 potentially relevant records across all databases, including duplicates. A total of 1003 duplicate records were removed, leaving 5113 records screened by title and abstract, of which 4002 were excluded. A total of 1111 full-text articles were retrieved for further review, and 1087 were excluded for not meeting the eligibility criteria. Twenty-four articles were included in the final synthesis as they addressed the barriers and facilitators of translating research evidence to action in Zambia. The Preferred Reporting Items for Systematic Reviews and Meta-Analyses (PRISMA) flow diagram is presented in Figure 1.

**FIGURE 1:**
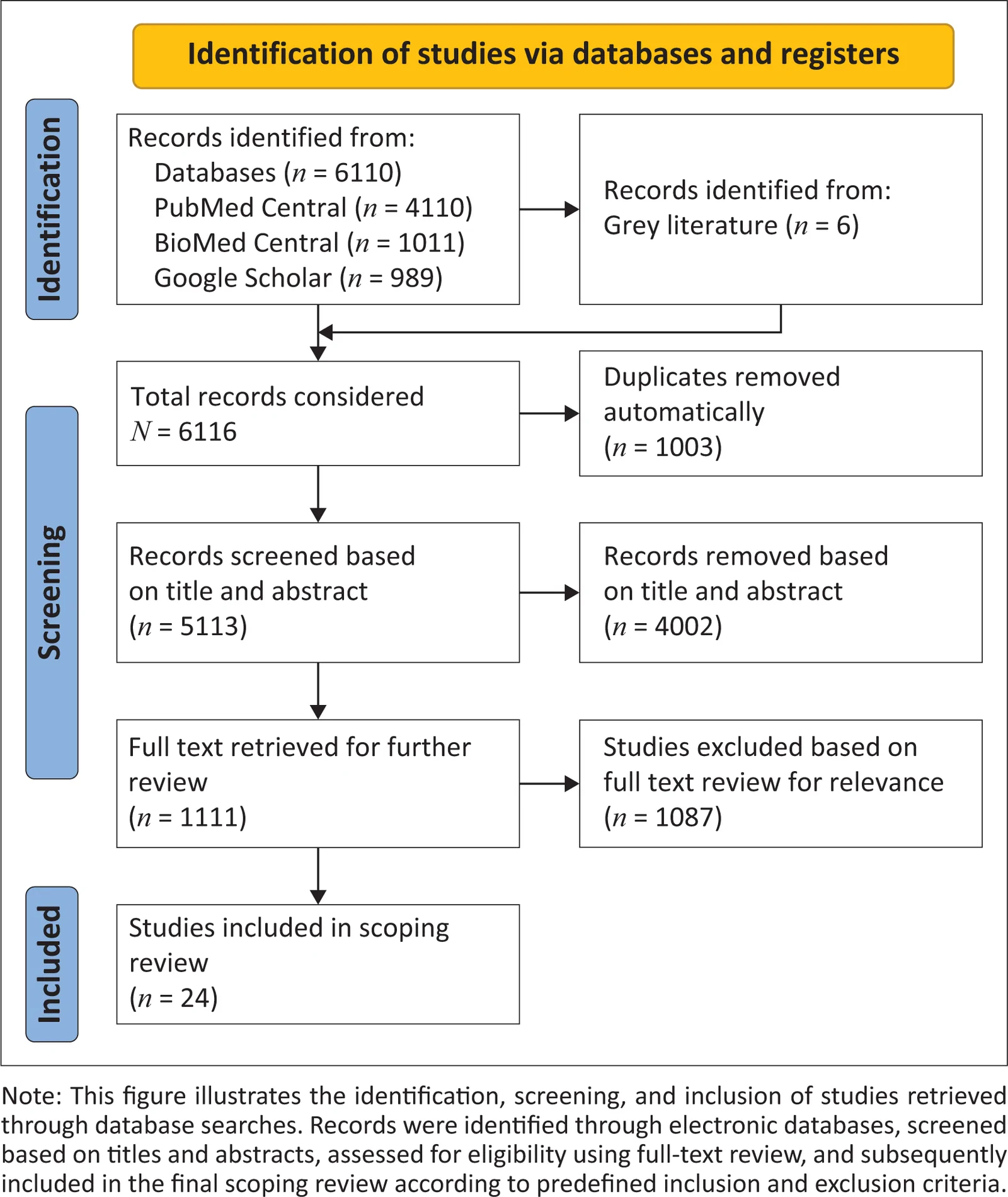
Preferred Reporting Items for Systematic Reviews and Meta-Analysis flow diagram of the study.

### Data extraction and synthesis

Data were entered into a manually developed data extraction sheet (Appendix 1), which was piloted on several articles and refined accordingly. A manual extraction sheet was used because of the small number of studies included in the review and to allow for flexibility in capturing the diverse formats of evidence included, ranging from peer-reviewed articles to grey literature and programmatic reports, which did not always conform to the structured fields of standardised digital tools.

### Data extracted included

Data extracted included author details, year of study, country, barriers and facilitators of KT, and recommendations. Two members of the research team independently performed data extraction and cross-checked entries for accuracy and completeness. Discrepancies were resolved through iterative discussion with reference to the research question and agreed eligibility.

### Data analysis

Data were analysed using manual thematic analysis guided by a deductive coding framework based on the taxonomy of barriers and facilitators to evidence use by policymakers developed by Oliver et al.^17^ This framework provided a structured approach for synthesising and presenting complex evidence to better support the policymaking process.^18^ Two team members independently applied the coding framework to data from the 24 included studies. Both recurring and newly identified themes were categorised accordingly. Factors which affected evidence use were coded as barriers or facilitators, against which were iteratively updated as new factors were identified, and the identified themes were validated via consensus. Additional themes that emerged during data analysis were categorised accordingly.

### Inclusion and exclusion criteria

The inclusion criteria comprised peer-reviewed articles, policy documents, reports and other relevant grey literature that were written in English and focused on KT processes, barriers, facilitators or frameworks within Zambia. Eligible studies included those examining health or public health research and its application in policy, practice or programme implementation.

The exclusion criteria covered articles not written in English, studies conducted outside Zambia or lacking relevance to the Zambian context, and publications that did not address KT and studies without accessible full texts.

## Review findings

We reviewed 24 studies from an initial collection of 6116 articles from PubMed Central, BioMed Central, Google Scholar and a direct Google search for grey literature (Figure 1).

### Barriers to knowledge translation in Zambia

Key themes that emerged as major barriers included inadequate relevant information for decision-making at different levels, poor access to information by policymakers, limited KT capacity, limited funding, weak relationships between researchers and policymakers, absence of national KT guidelines and the lack of central knowledge hubs. These are summarised in Figure 2 and below.

**FIGURE 2:**
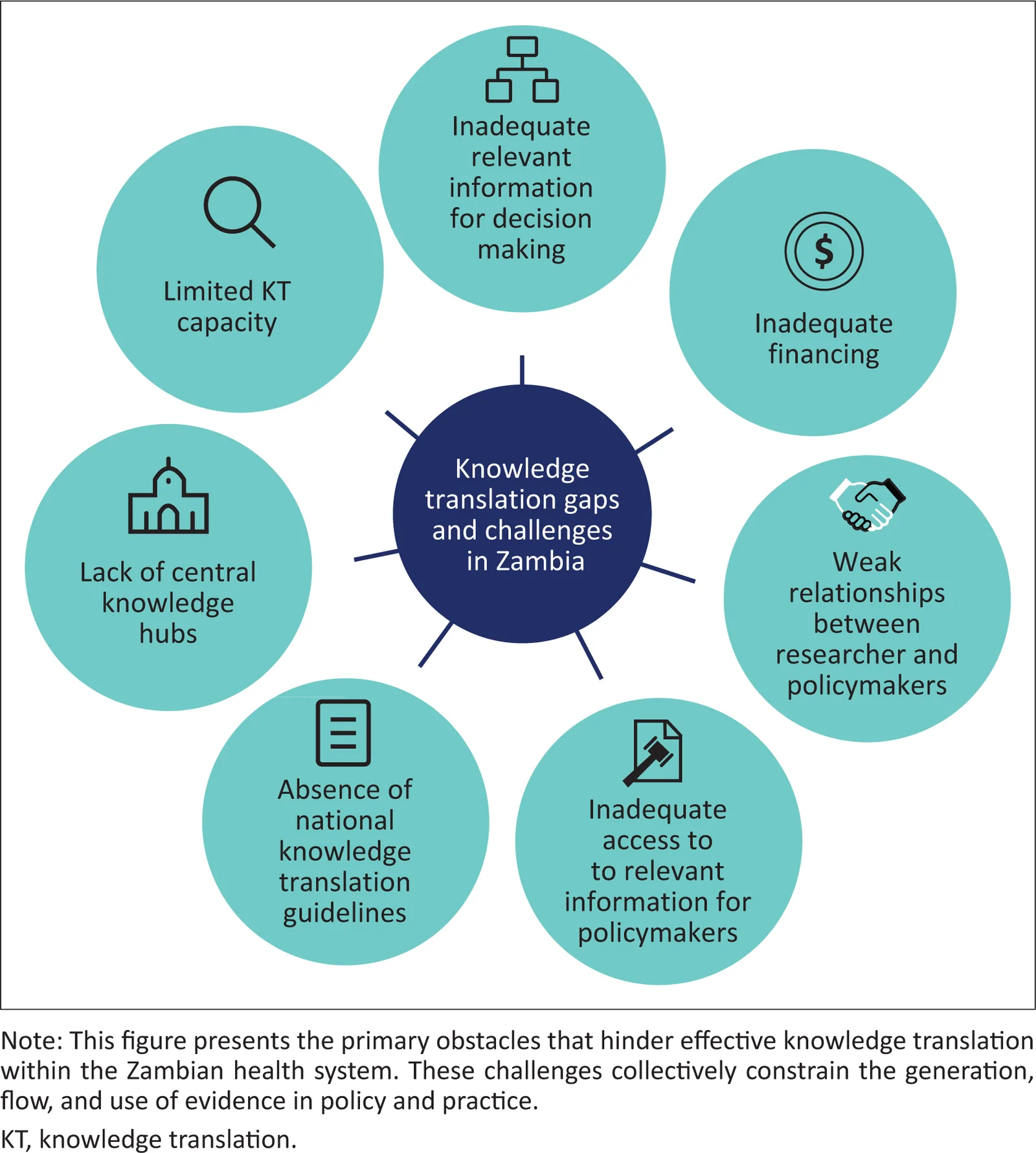
Major barriers to knowledge translation in Zambia.

#### Inadequate relevant information for decision-making

The lack of accurate, complete and timely data hindered KT because decision-makers did not have the evidence they needed to make decisions. When research did not address policy priorities, it reduced the likelihood of its being used.^18,19^ Furthermore, researchers often misunderstood policymakers’ needs, leading to low uptake of research evidence. For example, Katowa-Mukwato et al. found that evidence needs of decision-makers were not being met, resulting in low utilisation of research.^20^

#### Inadequate access to relevant information for policymakers

Policymakers often faced difficulties accessing relevant evidence because research was often scattered across multiple journals, repositories and institutions, making it difficult for policymakers to locate and synthesise relevant findings. Furthermore, there was poor access to good-quality relevant research because most research in LMICs was either externally driven or generalised from other settings, limiting applicability to the local context.

Furthermore, research remained inaccessible to policymakers because it was sometimes packaged and disseminated in formats that policymakers found difficult to use. Research was often packaged in technical formats, and even when published locally, policymakers did not have time to consult lengthy journals.^22,23,24^ For instance, a study by Banda et al.^21^ reported that the complexity of research papers and researchers’ lack of understanding of policy needs made it difficult for Zambian policymakers to interpret research evidence. Simplifying research outputs into easy-to-understand policy briefs, summaries or infographics was shown to improve uptake by policymakers.^25,26^

#### Limited knowledge translation capacity

Knowledge translation capability was defined as individuals’ knowledge and skills required to engage in translation practice.^27^ A lack of skilled personnel for KT and institutional capacity was another major barrier. Malama et al.^28^ reported that both researchers and policymakers in Zambia identified insufficient KT capacity as a critical gap.^28,20^

#### Inadequate financing

Funding constraints undermined KT activities in Zambia and remained a critical barrier across many studies in this review.^29,30,31^ The heavy dependence on donor funding limited sustainable KT processes in the country.^8^

#### Weak relationships between researchers and policymakers

Trust and communication gaps between researchers and decision-makers were significant barriers. Minimal engagement and trust limited KT activities, resulting in weak relationships between researchers and policymakers.^32,33^

#### Absence of national knowledge translation guidelines

The lack of a structured and coordinated system to implement, monitor and coordinate KT was a persistent key KT barrier.^34^ Although Zambia has established several structures to support KT, such as the National Health Research Authority (NHRA) mandate for KT and the Data-to-Policy (D2P) programme, these frameworks faced significant limitations. The lack of a clear national KT framework and guidelines was shown to limit effective coordination of evidence into policy and practice. The absence of mandated processes or guidelines means that evidence generation and use remain fragmented.^35^

#### Lack of central knowledge hubs

Limited platforms for research evidence synthesis, exchange and dissemination were major barriers to effective KT in Zambia. We found that structured platforms for evidence use were few; hence, strategic dissemination of research findings was limited to a few stakeholders, presentation formats were often inaccessible, and the overall visibility and accessibility of research outputs translated into policy and practice remained low. Furthermore, the lack of a centralised knowledge hub restricted access to national and subnational data needed for informed decision-making.^18,34,36^

### Facilitators of knowledge translation in Zambia

A review of the 24 included articles identified key facilitators of KT in Zambia such as supportive policy and legal frameworks, institutionalised governance structures for KT, capacity building, effective knowledge generation and dissemination platforms and strong stakeholder engagement and collaboration (Figure 3).

**FIGURE 3:**
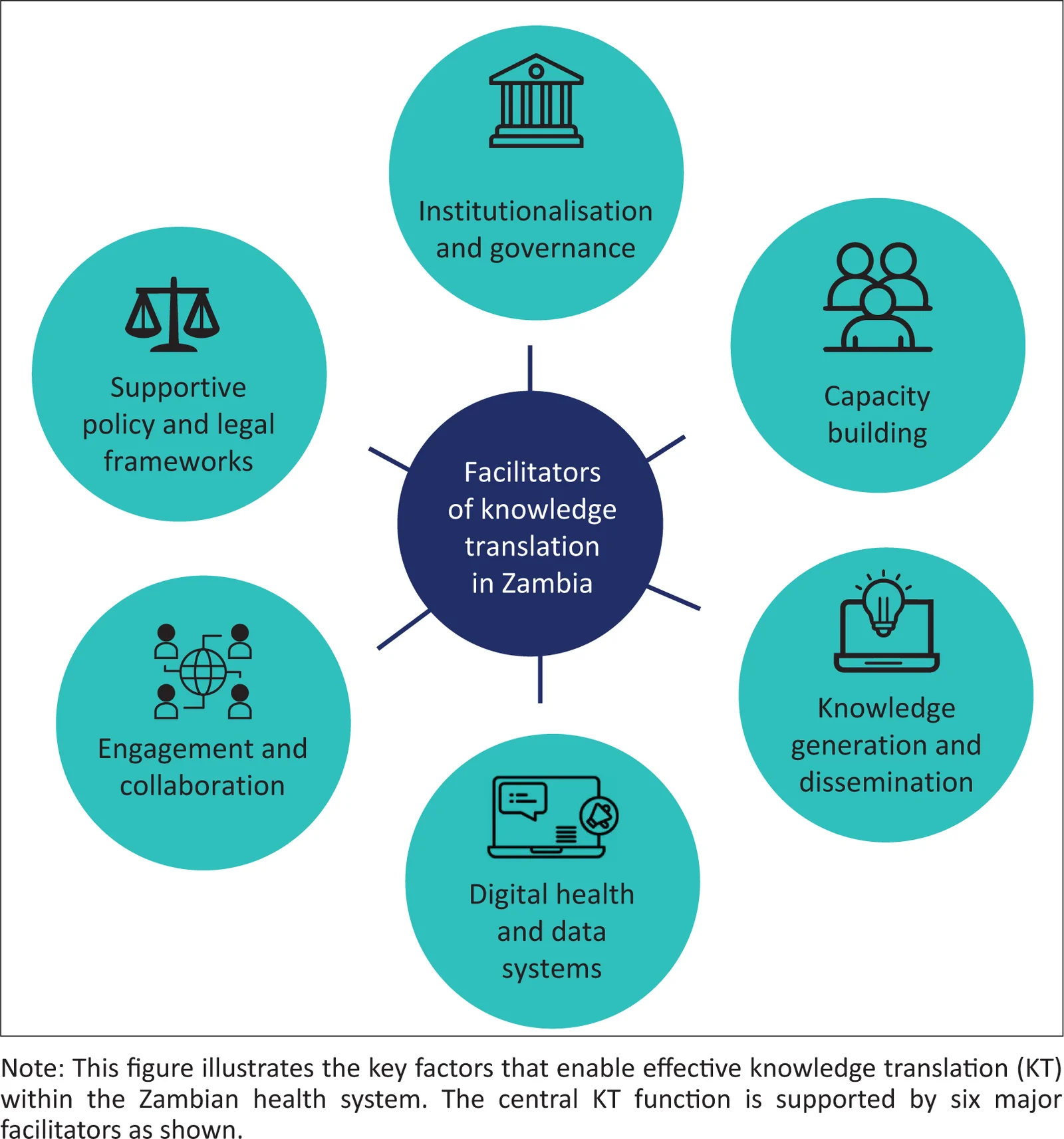
Facilitators of knowledge translation in Zambia.

#### Supportive policy and legal frameworks

This review identified supportive policy and legal frameworks. The *National Health Research Act of 2013* laid the legal foundation for the NHRA, which regulates and promotes health research. The National Health Research Agenda identifies priority research areas aligned with national needs and is a guide for both researchers and policymakers. The *National Research and Training Institute Act No. 13 of 2024* was enacted, marking an important reform in Zambia’s research governance system. This legislation restructures the national research framework by establishing the National Research and Training Institute to strengthen coordination, financing and capacity building in research across sectors. The Act further provides the legal and policy foundation for setting research priorities, regulating ethics and registration, and guiding the funding and coordination of research activities in the country.^37^

#### Institutionalisation and governance

With support from the Ministry of Health (MoH NHRA developed Terms of References [ToRs]) and facilitated the appointment of Research and KT Focal Point Persons (R&KT FPPs) in the 10 (*n* = 10) provinces in Zambia to spearhead research and KT activities. Consequently, with support from Centers for Disease Control and Prevention (CDC) Foundation, NHRA has engaged the R&KT FPPs in its research and KT training and mentorship programmes. The R&KT FPPs have been trained in Research Methods and Scientific Writing, as well as a KT D2P mentorship course.^29^

#### Capacity building

Significant efforts have been made to build KT capacity in Zambia through initiatives such as the D2P programme, which equips policymakers and health professionals with the skills to interpret and apply research evidence in decision-making. The D2P programme was launched in 2016 through the collaboration of the NHRA, the CDC Foundation and The Global Health Network as part of the Bloomberg Philanthropies Data for Health Initiative. According to the International Association of National Public Health Institutes (IANPHI) Data Impact report, Zambia has conducted five D2P training cohorts (2016, 2018, 2019, 2021 and 2022), which collectively resulted in the development of 16 policy briefs.^38^

Training in electronic medical health records, such as the District Health Information System 2 (DHIS2) for routine data use, significantly enhanced MoH’s capacity to collect, analyse and utilise health information for decision-making. These trainings not only focused on data entry and analysis but also built on competencies in data interpretation, visualisation and application of evidence for planning and policy formulation.^18^

#### Knowledge generation and dissemination

The scoping review found that Zambia had fostered platforms for knowledge exchange, which facilitated the translation of research into public health policy and practice and raised awareness of key evidence among policymakers and practitioners. Such platforms included the biannual research conferences organised by NHRA that allowed stakeholders to share findings. Policy briefs and journal publications were also used to disseminate evidence in accessible formats. Organisations like the Zambia Forum for Health Research (ZAMFOHR) provided forums for dialogue between researchers and policymakers.

#### Stakeholder engagement and collaboration

Inclusive engagement approaches emerged as facilitators. Policy laboratories and community advisory boards were identified as useful structures that could promote collaboration between researchers, policymakers and community members. These forums encouraged co-design of research and ensured community needs shaped research priorities. Increased recognition by decision-makers of research value, coupled with community engagement, helped align health interventions with local needs.

## Discussion

This scoping review aimed to identify the barriers and facilitators of translating research evidence into public health policy and practice in Zambia. The synthesis of 24 studies revealed that while important progress has been made through supportive policy instruments, institutionalisation of KT roles, and capacity-building programmes, systemic barriers still limit the consistent use of research evidence in decision-making.

The absence of national guidelines for KT has been consistently identified as a major barrier to evidence-informed decision-making, not only in Zambia but elsewhere as well.^39^ For most LMICs, the absence of clear KT structures within government ministries and research institutions contributes to duplication of efforts, poor information sharing and underutilisation of local evidence in national health planning.^35,40^ National KT guidelines can serve as a strategic framework to define clear processes for evidence generation, synthesis, dissemination and uptake in policy and practice. Several studies have indicated that without a standardised national framework to guide KT processes, the use of research evidence in policy and planning remains fragmented. A global systematic review found that fragmentation of KT interventions and activities remains a major challenge, emphasising the need for national guidelines to strengthen coordination and increase impact.^41^ Lack of coordination results in limited communication between researchers and policymakers.^21^

The absence of a national central knowledge hub for health research findings was also identified as a significant barrier to KT, contributing to information silos, duplication of research efforts and delays in evidence use.^17,42,43^ Currently, research outputs are spread out across various institutions and often inaccessible to policymakers who need timely evidence to guide decisions. The lack of timely research to support KT activities is sometimes because conducting rigorous studies can take years, which may not align with the fast pace of policy decision-making. A global systematic review by Oliver et al.^17^ cited poor access to good quality relevant research and the lack of timely research output as a major barrier to KT. Timely access to good-quality and relevant research evidence will promote KT activities.^17^

Establishing a national central knowledge hub would serve as an integrated platform where research findings are stored, tailored and disseminated in user-friendly formats. Such a hub would promote transparency, improve access to local and international evidence and facilitate collaboration among researchers, practitioners and policymakers. Evidence from other African countries, such as South Africa and Kenya, demonstrates that centralised research repositories enhance knowledge sharing and contribute to more coordinated and evidence-informed policy development.^8,17^ There are efforts by NHRA and the WHO to establish a repository for research and programme data in Zambia, which will lead to improved access to evidence for decision-making.

Another KT barrier was the lack of understanding of policy needs by researchers, along with the complex nature of research articles, which made it difficult for policymakers in Zambia to understand and translate research evidence into action.^21^ Presenting research findings in less complex formats, such as policy briefs, research summaries and infographics, has been shown to improve research uptake by policymakers.^25^ However, to achieve this compromise, researchers need to be trained in KT to enable them to have the skills to simplify scientific jargon. Other strategies, such as tailored messaging and context-specific KT interventions, have proved to be effective in enhancing uptake and application of research findings in decision-making.^26^

The scarcity of accurate, comprehensive and timely data aligned to the needs of policymakers hinders effective KT activities. Researchers don’t always align their studies with policymakers’ priorities, resulting in research outputs that are underutilised.^18,21^ Other studies done in LMICs also highlight similar challenges.^22,44^ Engaging policymakers early when conceptualising research questions can improve the relevance and uptake of evidence.^45^ If researchers and policymakers co-conceptualise research and policymakers are involved during implementation of the research, policymakers will likely have confidence in the research findings and become more willing to use it for policy formulation. This will also assist researchers in understanding the policymaking process, and on the other hand, policymakers will be kept aware of ongoing research.^33^

Inadequate funding for research and KT activities is another key barrier.^29,30,31^ African Union member states invest an average of 0.45% of gross domestic product (GDP) in research and development, against the 1% of GDP the African Union called on them to commit in 2007 and a global average of 1.7% (Africa CDC 2026).^46^ Health research specifically has fared no better: a decade after the 2008 Algiers Declaration committed governments to allocate at least 2% of national health expenditure to research (WHO Regional Office for Africa 2008),^47^ only 2 of 39 assessed countries in the WHO African Region had met the target (Nabyonga-Orem, Asamani & Makanga 2021).^48^ In addition, over 60% of research funding in SSA comes from external donors, with limited domestic investment in KT and implementation research.^49^ This situation has been further worsened recently by the United States (US) government funding cuts, which used to support the funding of health research in most SSA countries. To address this, African governments need to ensure that a dedicated budget for research and KT is prioritised, including support for local journals.^50,51^

In many African countries, limited domestic resources for research and KT activities force many researchers to rely on donor-driven agendas.^8^ In Zambia, despite the existence of a national research agenda, researchers are more inclined to conduct research based on donor rather than national preferences because of limited funding.^52^ In Uganda, insufficient funding meant that KT activities like dissemination workshops were not institutionalised, despite policymakers valuing research.^53^ Without adequate research funding, local priorities may go unstudied.^40^

Limited KT capacity among stakeholders was consistently reported as a barrier. A study by Malama et al. found that both Zambian researchers and policymakers lack sufficient KT training.^28,20^ A review by Edwards et al.,^54^ looking at studies in Africa, also noted insufficient KT skills in both researchers and policymakers. Building capacity through joint workshops, training and mentorship can empower researchers and policymakers to collaborate on evidence generation and use.^29,55,56^ For example, involving policymakers in research projects can help develop their ability to interpret evidence.^25,26,28^ Collaboration between researchers and policymakers can further build mutual capacity and trust.^55,56^

Weak relationships between researchers and policymakers were identified as another critical barrier. A study by Kasonde and Campbell found that a lack of trust and engagement between these groups hindered KT in Zambia.^32,33^ Power imbalances can undermine collaborative research; joint planning and co-creation are key.^57^ Recent case studies in Africa found that researchers often dominate processes, limiting co-production and ownership; thereby fostering collaborative relationships will improve KT.^57^

In Zambia, developing regular KT forums involving both researchers and policymakers could enhance mutual understanding.^58,59^ Such collaboration ensures research is informed by policy needs and that policymakers trust and use the evidence produced. Furthermore, structured interactions such as policy dialogues in Uganda have successfully built trust and improved evidence uptake.^37^ Zambia can learn from these examples by institutionalising regular engagement platforms to strengthen collaboration.^58,59^

Despite the aforementioned barriers to the translation of research evidence into policy and practice, we also identified positive things that were supportive of KT in Zambia. The enactment of the *National Health Research Act (2013)* and the *National Research and Training Institute Act (2024)* has provided a solid legal foundation for research regulation and coordination. These policies align with the WHO’s recommendation that national KT systems should be anchored in law and policy to ensure sustainability.^12^ The NHRA plays an important role in promoting evidence-informed decision-making by regulating, coordinating and supporting research that responds to research priorities. This policy environment mirrors regional best practices observed in countries like Kenya and Ghana, where legislative backing has strengthened KT governance.^53^

Capacity-building efforts have been instrumental in improving policymakers’ ability to interpret and apply research evidence. The NHRA’s establishment of provincial R&KT FPPs ensures decentralisation and consistency in KT implementation. Through support from various stakeholders, these focal persons have been trained in research methods, scientific writing and evidence-informed policymaking under the D2P programme. Similar governance models have been used in Uganda’s KT Platform and Malawi’s evidence-informed decision-making unit, both of which have significantly improved evidence use in health planning.^8,15^

The promotion of knowledge exchange through national research conferences, dissemination workshops and policy briefs has created structured spaces for interaction among various researchers and stakeholders. These structured platforms, organised by NHRA, provide a platform for evidence dissemination and encourage stakeholder collaboration and engagement. Regional experiences, such as Ghana’s Health Research Summits, show that such dissemination platforms significantly improve visibility and uptake of locally generated evidence.^53^

Overall, Zambia’s current KT status is consistent with the WHO’s Evidence, Policy, Impact framework,^12^ which emphasises engaging policymakers, building capacity and providing high-quality, usable evidence. The KT facilitators identified in this review reflect important enablers. However, without complementary investments (e.g. financing, guidelines and coordination), these will only partially mitigate the barriers to effective KT.^17^

### Limitations

This review had some limitations, such as methodological gaps. Many of the included studies relied on small, purposive samples drawn predominantly from national-level institutions in Lusaka, and most studies employed qualitative or descriptive designs. While these approaches are well-suited to exploring context and perceptions, they limit causal inference about which KT interventions work, for whom, and under what conditions. Few studies employed longitudinal or experimental designs, meaning that most evidence on barriers and facilitators reflects perceived rather than objectively measured KT outcomes. Geographically, the available evidence was heavily concentrated in urban and peri-urban settings, particularly Lusaka.

The Arksey and O’Malley’s framework and PRISMA-ScR captured the breadth of available evidence rather than methodological quality. Although the databases used were broad, they may not have captured all relevant indexed literature. The restriction to English-language publications may have excluded relevant studies, reports or institutional documents produced in other languages. Although grey literature was included, unpublished evaluations and internal reports held by the MoH, research institutions or implementing partners may contain evidence not captured here. Finally, data extraction relied on a manually developed sheet rather than a standardised digital platform, which, despite piloting and cross-checking, may have introduced some errors.

## Conclusion

Zambia has made measurable strides towards strengthening KT through supportive legislation, the appointment of provincial KT focal point persons, and capacity-building initiatives. Yet the absence of national KT guidelines and sustainable financing continues to limit the effectiveness of these efforts. The findings of this review point to several actions that can build on existing progress.

At the institutional level, Zambia needs a comprehensive national KT guideline. There is a need to ringfence dedicated domestic funding for both research and KT activities. Establishing a centralised knowledge hub for evidence dissemination and storage would address the fragmentation that currently hampers evidence uptake across health domains. Regular KT training for both researchers and policymakers, alongside structured platforms for their interaction, would strengthen the relational infrastructure that formal policies alone cannot substitute (see Appendix 2).

For policy, these findings underscore that legislation and structural commitments are necessary but insufficient without mechanisms to monitor KT outcomes and sustain stakeholder engagement. Policymakers should prioritise embedding KT functions within existing health governance structures rather than treating them as parallel activities. Future research should focus on evaluating the effectiveness of emerging KT platforms in Zambia, examining the role of power dynamics and political context in evidence uptake, and developing locally validated metrics for monitoring KT progress. Taken together, these steps can ensure that research informs decision-making more consistently and contributes to improved health outcomes across Zambia.

## Appendix 1

**TABLE 1-A1:** Data extraction sheet.

Number	Name of author	Year of publication	Country of focus	Type of study	KT gaps identified in summary
1	Malama et al.^27^	2021	Zambia	Exploratory qualitative study	Coordination and linkage of the knowledge creation, translation and policymaking processes remains a challenge Research knowledge translation capacity Limited resources Lack of a national knowledge hub for health research findings
2	Banda et al.^20^	2023	Zambia	Review	Lack of structured platforms for the use of evidence in policy planning Insufficient synthesis and exchange mechanisms for evidence Limited capacity among both researchers and policymakers to package and apply evidence Weak stakeholder coordination at local levels Researchers misunderstand the needs of decision-makers and, consequently, decision-makers may not use existing research evidence in their decision-making
3	Kasonde and Campbell^49^	2012	Zambia	Review	Limited resources for KT planning, staffing, capacity building, database maintenance, and stakeholder engagement Severe gaps in essential KT skills Difficulty in finding leaders with dual legitimacy in both research and policy realms
4	Katowa-Mukwato et al.^28^	2018	Zambia	Cross-sectional study	The concept of Evidence-Informed Health Policy was not well understood Low utilisation of evidence for decision-making
5	Maritim et al.^42^	2024	Zambia	A reflective commentary	Epistemic injustices Simplified conceptualisations of evidence-informed interventions Limited theorisation of the complexity of low-resource contexts and it impacts on implementation Persistent lags in transforming research into practice Limited focus on strategic dissemination of implementation science knowledge Existing training and capacity-building initiatives’ failure to engage a broad range of actors including practitioners through diverse learning models
6	Kwibisa^34^	2009	Zambia	Cross-sectional	The existing research agenda is outdated and presently, national priority setting mechanism is not well articulated and not clearly understood by researchers Inadequate financing from Government to support research, the absence of a mechanism to source for and secure funding from other sources Lack of institutional and human resource capacity to conduct research There is no elaborate system in place to monitor and coordinate research work
7	Parramore^18^	2017	Zambia	Interpretive case study	Lack of accurate, complete and timely data to facilitate the decision-making Inadequate capacity to analyse and use data for decision-making, as well as lack of standardised process for its usage Lack of data available at subnational level In low-recourse settings, decisions are often compromised by financial constraints
8	Maambo and Sakala^29^	2023	Zambia	Cross-sectional	Limited Funding and partnerships Lack of capacity to utilise evidence for decision-making
9	Moyo^39^	2021	Zambia	Cross-sectional	Inadequate research funding Sustainability of local journals Restrictions in the modes of dissemination research findings Lack of visibility and accessibility of research findings Restrictions in participating in setting of research agendas Lack of political will by the government Lack of national guidelines for research
10	Kapiriri and Chanda-Kapata^62^	2018	Zambia	Literature review and systematic facilitated workshop discussions	The limited capacity for the priority setting institutions to identify and fund their own research priorities Lack of conextualised research appropriate to the Zambian context
11	Chavula et al.^60^	2024	Zambia	Qualitative case study design	Inadequate coordination between researchers and decision-makers Lack of shared motivation and understanding Inadequate capacity for KT implementation
12	Cleaver et al.^35^	2020	Zambia	Cross-sectional	No clear structure for knowledge translation It is difficult to and maintaining stakeholder attention through all stages of the policy cycle
13	Gantayat et al.^30^	2024	Zambia	Cross-sectional	Staff attrition at ministries Lack of adequate and sustainable funding Lack of long term institutional capacity Low political support
14	Kuhrt et al.^63^	2025	Zambia and Sierra Leone	Cross-sectional	Evidence for decision-making is not available or provided in a timely manner Weak relationships between researchers and decision-makers
15	Moomba et al.^56^	2025	Zambia	Document review	Lack of adequate funding Not involving all the relevant stakeholders
16	Mukamba et al.^43^	2020	Zambia	Cross-sectional	Lack of access to of evidence for decision-making Inaccurate data for decision-making Presentation of information in a format that is not easy to understand
17	Chilengi-Sakala et al.^65^	2023	Zambia	Review	Lack of adequate knowledge translation capacity Weak partnerships between researchers and policymakers
18	Ashraf et al.^44^	2011	Zambia	Randomised field study	Limited evidence on how to select and motivate community-based agents to distribute health products Lack of knowledge on the relative effectiveness of monetary vs. non-monetary incentives in promoting sustained community health engagement
19	The Zambia Forum for Health Research^64^	2008	Zambia	Descriptive case study	Research outputs often remain inaccessible and fail to inform decision-making Much research conducted in Zambia does not reach policymakers or practitioners
20	Hanefeld and Musheke^61^	2008	Zambia & South Africa	Qualitative cross-sectional policy implementation study	Limited evidence on the subnational impact of Global Health Initiatives (GHIs) Weak accountability of GHIs at country level in terms of governance and integration into local systems
21	Matenga et al.^32^	2019	Zambia	Qualitative cross-sectional study	Limited documentation of the voices and experiences of African researchers in partnerships Lack of attention to how partnership guidelines are applied in practice Gaps in equity and ownership of research agenda, samples, and data Power imbalances driven by the funding structure
22	Lee et al.^36^	2023	Zambia	Qualitative cross-sectional study	Unclear accountability, limited authority at facility or district level Long distances between the facilities and the district health offices cause challenges with submitting monthly paper-based reports Lack of clarity on whether HMIS data meet the practical needs of end-users
23	Kivu International^57^	2024	Zambia	Descriptive case study	Weak link between economic or policy evidence and political decision-making. Lack of policy alternatives grounded in both technical evidence and political feasibility. Limited ability of fragmented CSOs to influence without coalition building.

Note: Please see the full reference list of the article Shanaube K, Chilengi-Sakala S, Banda K, et al. Barriers and facilitators of translating research evidence into public health policy and practice in Zambia: A scoping review. J Public Health Africa. 2026;17(1), a1837. https://doi.org/10.4102/jphia.v17i1.1837, for more information.

KT, knowledge translation; HMIS, Health Management Information System; CSO, Central Statistics Office.

## Appendix 2

**TABLE 1-A2:** Summary of knowledge translation policy recommendations and responsible stakeholders in Zambia.

Recommendation	Responsible stakeholders
1. Develop and implement a National Knowledge Translation (KT) Framework	Ministry of Health (MoH), National Health Research Authority (NHRA)
2. Create national guidelines for KT	NHRA, Ministry of Health (MoH)
3. Establish a centralised knowledge hub for evidence dissemination and storage	NHRA
4. Increase dedicated domestic funding for KT activities	Ministry of Finance, MoH, Parliament Budget Committee
5. Provide KT training for researchers and policymakers	NHRA, Academic Institutions, Research Organisations
6. Creating platforms for regular researcher and policymaker interaction.	Researchers and policymakers

Note: This table outlines key recommendations for strengthening knowledge translation (KT) in the national health system and identifies the primary stakeholders accountable for implementing each action. Stakeholders include government ministries, regulatory bodies, academic institutions, and research organisations responsible for leading or supporting the proposed KT initiatives.
